# The Role of Zinc in Pediatric Asthma and Allergic Rhinitis: Mechanisms and Clinical Implications

**DOI:** 10.3390/nu17162660

**Published:** 2025-08-17

**Authors:** Giulio Dinardo, Cristiana Indolfi, Angela Klain, Carolina Grella, Maria Angela Tosca, Eleonora Ruocco, Michele Miraglia del Giudice, Giorgio Ciprandi

**Affiliations:** 1 Department of Woman, Child and General and Specialized Surgery, University of Campania “Luigi Vanvitelli”, 80138 Naples, Italy; dinardogiulio@gmail.com (G.D.); cristianaind@hotmail.com (C.I.); caro.grella94@gmail.com (C.G.); michele.miragliadelgiudice@unicampania.it (M.M.d.G.); 2 Allergy Center, IRCCS Istituto Giannina Gaslini, 16147 Genoa, Italy; mariangelatosca@gaslini.org; 3 Dermatology Unit, Department of Mental and Physical Health and Preventive Medicine, University of Campania “Luigi Vanvitelli”, 80138 Naples, Italy; eleonora.ruocco@unicampania.it; 4 Allergy Clinic, Casa di Cura Villa Montallegro, 16145 Genova, Italy; gio.cip@libero.it

**Keywords:** zinc, asthma, rhinitis, allergic children, trace elements, oxidative stress, hypersensitivity, zinc supplementation

## Abstract

Pediatric asthma and allergic rhinitis are prevalent chronic inflammatory diseases ruled by complex interactions among genetic, environmental, and nutritional factors. Zinc, an essential trace element, plays a crucial role in immune modulation, oxidative stress regulation, and epithelial barrier maintenance, all of which are significant in the context of allergic airway diseases. This review aimed to explore and synthesize current evidence on the biological mechanisms and clinical implications of zinc in pediatric asthma and allergic rhinitis. A comprehensive literature search was conducted through PubMed and the Cochrane Library for studies published between 2015 and 2025. Eligible studies included observational and interventional research focused on zinc status or supplementation in children with asthma or allergic rhinitis. Numerous observational studies and meta-analyses indicated reduced circulating zinc levels in children with asthma, often correlating with poor symptom control, increased oxidative stress, and lower pulmonary function. In allergic rhinitis, zinc depletion in nasal mucosa was associated with elevated local inflammation, although paradoxical increases in zinc concentrations have been observed in nasal secretions during active disease. Interventional trials in pediatric asthma populations showed that zinc supplementation may improve clinical symptoms, reduce inflammation, and enhance lung function, although the results were inconsistent and limited by methodological variability. In conclusion, zinc plays a multifactorial role in modulating immune responses and maintaining mucosal health in pediatric allergic airway diseases. While zinc supplementation holds promise as a safe and accessible adjunctive therapy, further high-quality randomized controlled trials are needed to define its clinical utility and establish evidence-based guidelines.

## 1. Introduction

Pediatric asthma and other allergic diseases are among the most prevalent chronic conditions worldwide, affecting both children and adults [1]. According to recent estimates from the World Health Organization, asthma alone afflicted approximately 262 million individuals globally in 2019, accounting for 455,000 deaths yearly, and it was recognized as one of the top fifteen causes of years lived with disability [2,3]. In developed countries, the prevalence of asthma and allergic conditions such as allergic rhinitis and atopic dermatitis ranges between 10% and 25%, translating into substantial morbidity and considerable healthcare and societal costs [4]. In the United States, nearly 25 million people, about 13% of the population, have asthma, with an estimated annual economic burden of USD 56 billion [5]. Asthma occurs more frequently among children, with approximately 6.5% of those aged 6 to 11 years affected during their school years [1]. In Europe, over 128 million individuals are affected by allergic diseases, with up to 30% of younger Europeans reporting at least one allergic condition [6,7]. Simultaneously, developing nations are experiencing a worrisome rise in allergy prevalence, likely driven by urbanization, changes in hygiene practices, environmental pollution, and the “Westernization” of dietary patterns [6]. Since asthma has no definitive cure, management focuses on effectively controlling symptoms, preserving normal activity levels, reducing the likelihood of future exacerbations, and minimizing treatment-related side effects [6].

### 1.1. Dietary Interventions in Pediatric Allergic Diseases

Over the past decade, increasing attention has been given to the potential role of various micronutrients and food-derived compounds in the prevention and management of allergic diseases, including asthma and allergic rhinitis [8]. Traditional therapies, such as inhaled corticosteroids, antihistamines, and allergen avoidance, while effective in many cases, often offer only symptomatic relief and are limited by adherence issues, side effects, or incomplete disease control [9]. As a result, there has been a growing interest in integrative strategies that include nutritional interventions aimed at modulating immune function and maintaining mucosal barrier integrity [8]. Among these, trace elements such as zinc, iron, and selenium, along with vitamins A, C, D, and E, have been studied for their immunomodulatory and antioxidant properties. Deficiencies in these micronutrients are commonly reported in children with atopic conditions and have been associated with an altered Th1/Th2 balance, increased oxidative stress, impaired epithelial repair, and greater disease severity [10]. Notably, inadequate zinc and vitamin A intake have been linked to increased airway inflammation, while vitamin D deficiency may exacerbate Th2-mediated responses and lower asthma control [11]. In addition to classical micronutrients, bioactive compounds of natural origin have also shown promising potential. Lactoferrin, a multifunctional iron-binding glycoprotein found in human and bovine milk, has demonstrated antioxidant, antimicrobial, and anti-inflammatory effects and may help modulate allergic responses by regulating Th2 cytokines and suppressing mast cell degranulation [12]. Similarly, resveratrol, a polyphenolic compound naturally present in grapes, berries, and peanuts, has been investigated for its immunoregulatory and anti-inflammatory effects in respiratory diseases [13]. When administered in combination with carboxymethyl-β-glucan, an immunostimulatory polysaccharide, intranasal resveratrol has shown efficacy in reducing nasal symptoms in children with allergic rhinitis and in improving respiratory outcomes in children with recurrent wheezing and non-atopic asthma [14]. Clinical trials evaluating this combination have also reported reductions in school absences, medication use, and healthcare visits, indicating a significant impact on disease burden and quality of life [14,15].

### 1.2. Zinc Mechanism

Zinc is the second most abundant trace metal in mammals, with a total body content of approximately 2–4 g. It is found ubiquitously, with the highest concentrations in muscle (59% of total), bone (29%), skin (6%), and liver (5%). Zinc participates in over 300 enzymatic reactions, modulates gene transcription, and stabilizes protein structures [16,17,18,19]. In the immune system, zinc is vital for thymic hormone (thymulin) activity, T-cell differentiation, and the maintenance of immune tolerance [3]. Zinc deficiency, estimated to affect up to 20% of the global population, presents clinically with non-specific symptoms such as growth retardation, mental disturbances, frequent infections, impaired wound healing, and dysregulated immune responses [16,20]. Physiological serum zinc concentrations in healthy individuals typically range from 80 to 120 µg/dL. Zinc deficiency is generally defined as serum levels below 70 µg/dL in adult females, 74 µg/dL in adult males, and, for pediatric populations aged ≥10 years, below 66 µg/dL for girls and 70 µg/dL for boys [21]. These thresholds, however, are subject to variation based on factors such as age, sex, circadian rhythms, and the presence of inflammatory states; therefore, clinical interpretation should be contextualized with patient-specific risk factors and clinical findings [22].

**Figure 1:**
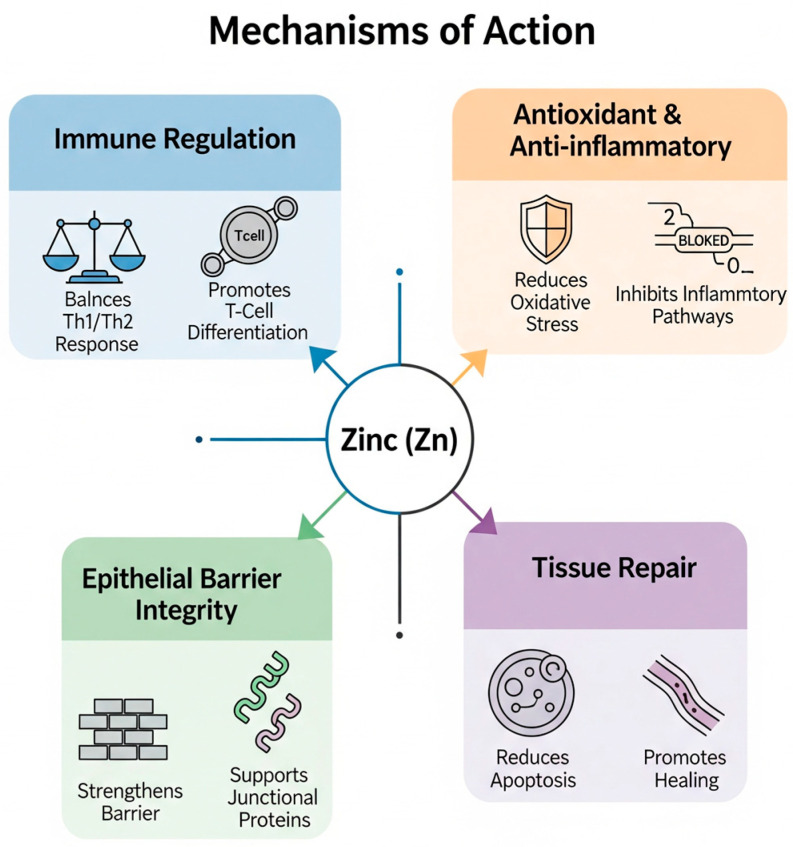
Mechanisms of action of zinc.

At the molecular level, zinc exerts a wide range of effects on allergic and infectious processes through several tightly interconnected pathways [18]. One of its most crucial functions is maintaining redox homeostasis. Zinc competes with redox-active transition metals, such as iron and copper, thereby limiting the generation of hydroxyl radicals [20]. Additionally, it serves as a crucial cofactor for copper−zinc superoxide dismutase (Cu/Zn SOD), an enzyme that converts superoxide anions into less reactive molecules, such as oxygen and hydrogen peroxide [18,20]. This antioxidant activity helps prevent lipid peroxidation within cell membranes, ultimately reducing the concentration of oxidative stress biomarkers, such as 8-iso-prostaglandin F2α (8-iso-PGF_2_α), which is known to promote the release of pro-inflammatory cytokines and the recruitment of immune cells in the airways [23]. Zinc also plays a crucial role in regulating the balance between Th1 and Th2 immune responses [24]. Its deficiency is associated with thymic atrophy and diminished thymulin activity, both of which impair T cell development and skew CD4⁺ T-cell differentiation toward a Th2-dominant phenotype. A key feature of asthma is an overactive Th2 immune response to environmental antigens that are typically non-threatening. Th2 lymphocytes release pro-inflammatory cytokines that drive allergic inflammation and enhance B cell activation, leading to the production of antibodies, particularly IgE [25,26,27]. This immune deviation leads to the increased secretion of interleukins, such as IL-4, IL-5, and IL-13, elevated IgE production, and enhanced eosinophilic activation, hallmarks of allergic inflammation [28]. Experimental studies have shown that low zinc levels can exacerbate airway eosinophilia by up to 35%, while zinc supplementation appears to counteract this effect and reduce the inflammatory burden [25,26,27]. The influence of zinc extends to innate immunity as well. In particular, it modulates the activity of group 2 innate lymphoid cells (ILC2s), which are stimulated by epithelial-derived alarmins and contribute to type 2 inflammatory responses in the airways. Zinc regulates both the proliferation and cytokine output of ILC2s [29]. Furthermore, it is involved in maintaining the homeostasis of dendritic cells (DCs). Zinc-responsive proteins such as the ubiquitin-editing enzyme A20 help control DC maturation and prevent excessive antigen presentation. When zinc is deficient, dendritic cells become hyperactive and lose their tolerogenic phenotype, resulting in exaggerated Th2 polarization and allergic sensitization [29]. Another essential function of zinc is the preservation of epithelial barrier integrity [29]. Zinc supports the expression and stability of key junctional proteins, including tight junction components such as claudins and occludin, as well as adherens junction molecules like E-cadherin [29]. The presence of extracellular zinc activates the GPR39 receptor, initiating a signaling cascade that involves phospholipase C, intracellular calcium release, and AMPK activation. This cascade promotes the assembly of junctional complexes and enhances barrier function. In contrast, zinc deficiency disrupts this process, resulting in increased degradation of junctional proteins, epithelial cell apoptosis, and compromised barrier integrity. Consequently, allergens can penetrate more easily, exacerbating immune responses and perpetuating chronic inflammation [3,29]. Finally, zinc exerts anti-apoptotic and tissue repair functions [30]. By inducing the expression of metallothioneins, zinc can inhibit the activation of caspases, thereby reducing epithelial cell apoptosis triggered by oxidative or toxic stimuli [20,30]. Additionally, zinc facilitates wound healing by modulating matrix metalloproteinase activity and promoting the migration and proliferation of keratinocytes. These actions not only protect airway integrity but also support the resolution of inflammation and the restoration of tissue homeostasis after injury (Figure 1) [31].

### 1.3. Rationale and Objectives

The multifaceted biological roles of zinc, including its contributions to antioxidant defense, immune system modulation, and epithelial barrier maintenance, provide a strong mechanistic basis for its potential involvement in the pathophysiology of atopic diseases, such as asthma and allergic rhinitis [3,28,32]. Observational studies have explored this link, with some reporting lower zinc levels in pediatric patients that correlate with poorer clinical outcomes [16]. However, these findings are not uniform across all populations and study designs; other investigations have found no significant association between systemic zinc status and the presence or severity of disease. This inconsistency in the clinical evidence, coupled with variability in assessment methods, complicates the interpretation of zinc’s role and its potential therapeutic utility [33]. Therefore, a comprehensive and critical synthesis of the available literature is required to explore these conflicting results. The objective of this review is to delineate the established mechanistic underpinnings of zinc’s actions, critically appraise the current and often contradictory clinical evidence from studies on pediatric asthma and rhinitis, and synthesize these findings to provide a clear overview of the state of the research and to propose precise directions for future studies.

## 2. Materials and Methods

**Figure 2:**
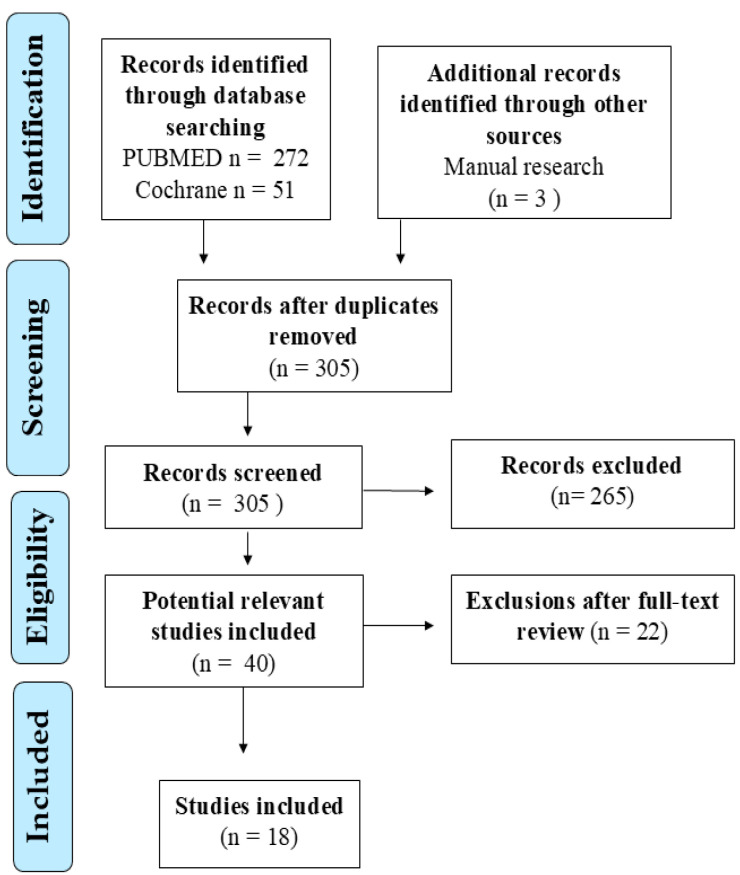
Summary of the literature search and study selection process.

An extensive bibliographic search was conducted using PubMed and the Cochrane Library to identify studies published in English between 2015 and 2025 on the role of zinc in pediatric asthma and allergic rhinitis. Both observational and interventional studies involving human subjects were considered. The search strategy was built using specific MeSH terms and related keywords. For asthma, the query was: ((“zinc” [MeSH Terms] OR “zinc” [All Fields]) AND (“asthma” [MeSH Terms] OR “asthma” [All Fields] OR “asthmas” [All Fields] OR “asthmas” [All Fields])) AND (y_10[Filter]). For allergic rhinitis, the following string was applied: ((“zinc” [MeSH Terms] OR “zinc” [All Fields]) AND (“rhinitis” [MeSH Terms] OR “rhinitis” [All Fields] OR “rhinitides” [All Fields])) AND (y_10[Filter]). Only studies involving children (0–11 years) or adolescents (12–18 years) with reported data on zinc status, deficiency, or supplementation were included. Articles focusing exclusively on adults or unrelated outcomes were excluded. After duplicate removal, all records were screened by title and abstract. One study was excluded due to retraction and another due to unavailability of the full text. Two reviewers (G.D. and C.I.) independently assessed the relevance and eligibility of each study. Disagreements were resolved through discussion with a third reviewer (M.M.d.G.). Full-text articles meeting inclusion criteria were reviewed in detail, and key data were extracted regarding the study design, participant characteristics, zinc measurement or intervention methods, and clinical or immunological outcomes. Clinical outcomes included scores obtained through questionnaires administered to patients to assess asthma control, numerical values of pulmonary function parameters (such as FEV_1_ and PEF), the frequency of exacerbations, and the presence as well as the quantification of any hospitalizations. Immunological outcomes included the analysis of cytokine profiles (such as IL-4, IL-5, IL-10, IFN-γ), eosinophil counts, and serum IgE levels, generally associated with asthma and rhinitis phenotypes mediated by TH2 responses (Figure 2).

## 3. Results

### 3.1. Zinc in Clinical Studies on Asthma

The investigation into zinc’s role in pediatric asthma reveals several key themes, as follows: the overall zinc status in affected children compared to controls, the influence of dietary zinc, correlations between zinc levels and clinical asthma parameters, and outcomes of interventional studies (Table 1) [3,10,27,28,33,34,35,36,37,38,39,40,41,42,43,44,45].

### 3.2. Zinc Status in Children with Asthma

Multiple systematic reviews and meta-analyses indicated altered zinc homeostasis in asthmatic children. A comprehensive meta-analysis by Chen et al. (1027 cases, 2150 controls) found significantly lower circulating zinc in asthmatics (SMD = −0.40; 95% CI −0.77 to −0.03), a finding that persisted after outlier exclusion (SMD = −0.26; 95% CI −0.40 to −0.13) and was consistent across various subgroups [34]. Similarly, Xue et al.’s meta-analysis of 21 articles (2205 participants) reported lower circulating zinc in children with asthma or wheezing versus healthy controls (SMD = −0.38; 95% CI: −0.60 to −0.17), with a more pronounced association in formally diagnosed asthma (SMD = −0.41) and in studies from Middle Eastern countries [35]. Several cross-sectional studies substantiated these findings. Kuti et al. documented significantly lower mean serum zinc in 80 Nigerian children with asthma compared to 80 controls (71.0 ± 30.3 µg/dL vs. 84.2 ± 31.7 µg/dL, *p* = 0.008) [36]. Srivastava et al. also identified lower serum zinc levels in 100 asthmatic children compared to 75 healthy peers (mean: 51 µg/dL vs. 60 µg/dL) [37]. Andino et al. found significantly lower median serum zinc levels in 24 urban children with moderate to severe persistent asthma on inhaled corticosteroids compared to controls (*p* = 0.0111). However, none met the criteria for clinical deficiency [38]. On the other hand, the evidence was not entirely uniform. Ghaffari et al.’s systematic review reported no significant difference in pooled mean serum zinc concentrations between asthmatic children and controls despite high heterogeneity (*I*^2^ = 96.1%). Nevertheless, the same review noted consistently lower hair zinc levels in asthmatics (*I*^2^ = 95.6%) [33]. Supporting the findings of no significant difference in serum zinc, AbdulWahab et al. found no disparity in mean serum zinc between 40 asthmatic and 40 healthy Qatari school-aged children (12.78 ± 1.8 µmol/L vs. 13.0 ± 1.52 µmol/L) [39].

### 3.3. Relationship Between Zinc Levels and Asthma Control

The relationship between zinc status and asthma’s clinical and physiological parameters revealed complex associations [46]. Rajkumar et al. found significantly higher serum zinc levels in those with well-controlled asthma compared to those with poor control (158.06 µg/dL vs. 129.23 µg/dL, *p* = 0.006) and a weak positive correlation between serum zinc and ACT scores (ρ = 0.26, *p* = 0.031). However, zinc levels did not vary by disease severity in this cohort [27]. In the study by Siripornpanich et al., plasma zinc levels were positively correlated with FEV_1_ and the FEV_1_/FVC ratio. Srivastava et al. observed the lowest serum zinc levels in children with uncontrolled asthma (49 µg/dL) and noted that lower selenium and vitamin D3 levels also correlated with worse asthma control [40]. A review by Maywald et al. consolidated evidence linking reduced zinc in serum or sputum to markers of oxidative stress, airway inflammation, more severe disease, increased exacerbations, decreased lung function, and elevated IgE in children [3]. However, some studies have not consistently found these associations. Kuti et al. reported that while moderate-to-severe asthma was associated with lower selenium levels, zinc levels did not differ significantly by severity [36]. Abdul Wahab et al. also found no association between serum zinc levels and asthma control, inhaled corticosteroid dosage, lung function, or total IgE [39]. Andino et al. reported reduced visual contrast sensitivity in asthmatic children (*p* < 0.01) but found no direct correlation with zinc levels. They hypothesized that lower zinc levels might be a consequence of chronic inflammation [38]. Peroni et al.’s review contextualized these findings by explaining that micronutrient deficits, including zinc, can skew immune responses towards a Th2-dominant profile and enhance mast cell reactivity, noting common deficiencies of iron, zinc, and vitamins A and D in children with atopic conditions [10].

### 3.4. Clinical Impact of Zinc Supplementation in Pediatric Asthma

Interventional studies and reviews assessing zinc supplementation suggest therapeutic potential. Rerksuppaphol et al. conducted an RCT in 42 children hospitalized for acute asthma exacerbation; zinc-treated children (over half of whom were zinc-deficient at admission) experienced significantly greater reductions in pediatric respiratory assessment measure (PRAM) at 24 h (*p* = 0.015) and 48 h (*p* = 0.042) compared to placebo, alongside a more substantial rise in serum zinc, with comparable safety profiles [41]. Ghaffari et al.’s systematic review, although noting the limited number of trials, reported that all three intervention studies found that zinc administration improved clinical symptoms and pulmonary function in asthmatic children [33]. Cheng et al., analyzing NHANES data (4597 overweight or obese U.S. youth), found an inverse association between higher dietary zinc intake (quartiles from ≤5.68 to ≥11.96 mg/day) and asthma prevalence in overweight or obese children and adolescents [42]. Maywald et al.’s review further stated that zinc supplementation generally appears to improve wheezing, cough, dyspnea, and lung function despite outcome variability across trials [3]. The same review highlighted the importance of maternal zinc intake, linking adequate levels to improved offspring lung function and a lower incidence of asthma. At the same time, deficiency was associated with an increased risk of bronchial hyperreactivity [3]. Animal data referenced therein showed zinc deprivation amplified bronchopulmonary eosinophilia, an effect countered by supplementation. The broader nutritional context was emphasized by Peroni et al., who noted that interventions with multiple micronutrients have shown reductions in atopic symptoms [10]. Xue et al.’s meta-analysis also proposed that zinc’s role in modulating immune responses and oxidative stress means its deficiency may disrupt the Th1/Th2 balance and exacerbate airway inflammation, cautioning about interactions with other micronutrients, such as iron and copper [35].

### 3.5. Zinc in Clinical Studies on Rhinitis

The investigation into the role of zinc in rhinitis reveals a complex interplay between systemic and local zinc homeostasis, inflammation, and clinical symptoms, with evidence drawn from human observational studies, animal models, and genetic analyses (Table 1).

### 3.6. Zinc Homeostasis in Rhinitis: A Localized Paradox

According to a prospective follow-up study by Xu et al. on patients with Japanese cedar pollinosis (JCP), zinc homeostasis is paradoxical during active inflammation [43]. They reported that during the pollen season, patients’ serum zinc levels significantly decreased compared to both the preseason period and healthy controls. Conversely, zinc levels in the nasal epithelial lining fluid (ELF) of these same patients significantly increased after pollen exposure, becoming markedly higher than in controls. This fact suggests a localized shift of zinc from the systemic circulation to the site of allergic inflammation. This phenomenon was successfully replicated in their corresponding JCP mouse model, which showed increased zinc in nasal ELF and decreased zinc in serum and nasal mucosa following the allergen challenge [43]. A similar paradox was observed by Suzuki et al. in the context of chronic rhinosinusitis with nasal polyps (CRSwNP). While they found no significant differences in serum zinc levels between patient groups and controls, they noted that zinc levels in mucus collected directly from inflammatory sites were significantly elevated [47]. In stark contrast, the nasal mucosal tissue of CRSwNP patients showed considerably decreased labile zinc levels compared to controls [42]. In a different approach, a Mendelian randomization study by Changhai et al., designed to investigate causal relationships, did not find evidence for a causal link between genetically predicted serum zinc levels and the risk of allergic rhinitis. That study, however, did find that genetically predicted higher serum selenium levels were associated with a reduced risk of AR [45]. This apparent redistribution of zinc during allergic inflammation may represent an adaptive, tissue-specific response aimed at mitigating local oxidative stress and promoting epithelial repair. Zinc is essential for maintaining mucosal barrier integrity and modulating immune cell activity; thus, its mobilization to the nasal mucosa might reflect an upregulation of local zinc transporters or binding proteins such as metallothioneins in response to allergen-induced epithelial damage. Furthermore, inflammatory mediators, including IL-6 and TNF-α, have been shown to regulate zinc transporter expression (e.g., ZIP8, ZIP14), which could facilitate localized zinc accumulation in the extracellular environment. Another plausible mechanism involves the recruitment of immune cells like neutrophils and macrophages to the inflamed mucosa, where zinc is released through degranulation or cellular turnover. The simultaneous depletion of serum and tissue-resident zinc pools, as seen in murine models, supports the hypothesis of compartmental redistribution rather than overall zinc excess. Further studies are warranted to clarify whether this localized zinc shift plays a protective or pathological role in allergic rhinitis pathophysiology.

### 3.7. Correlation with Pathophysiological Features

The clinical relevance of this altered zinc homeostasis was detailed in the work of Suzuki et al. on CRSwNP [47]. Their investigation revealed that the observed depletion of zinc in mucosal tissue was significantly correlated with key pathophysiological features of the disease. Specifically, they found a significant negative correlation between mucosal zinc levels and the number of infiltrating eosinophils, a hallmark of Th2-driven inflammation. Furthermore, lower mucosal zinc was significantly correlated with reduced collagen content, suggesting a role in adverse tissue remodeling [47]. The in vitro experiments from this study provided a functional link to these observations; according to Suzuki et al., zinc depletion of primary human nasal epithelial cells (HNECs) induced the production of pro-inflammatory cytokines IL-6 and IL-8, as well as MUC5AC, a key mucin gene. Their work also demonstrated that in primary human nasal fibroblasts, zinc depletion significantly reduced cell viability and the synthesis and secretion of collagen I [47].

### 3.8. Effects of Zinc Supplementation on Animal Models

Interventional studies in animal models provide strong evidence for the therapeutic potential of zinc. According to Xu et al., the direct intranasal application of zinc in a mouse model of JCP significantly alleviated allergic symptoms [43]. Mice treated with intranasal zinc plus an allergen showed an approximately 42% reduction in sneezing frequency and a 24% reduction in nose-rubbing behavior compared to mice treated with the allergen alone. This treatment also resulted in a significant decrease in the number of mucin-secreting goblet cells in the nasal mucosa [43]. Complementing these findings, a study by Shi et al. using an ovalbumin-induced AR mouse model demonstrated that dietary zinc supplementation effectively modulates key allergic markers [44]. They found that mice with zinc deficiency had significantly higher concentrations of total and allergen-specific IgE, which were reversed by zinc supplementation. This oral supplementation also reduced the elevated serum levels of the pro-inflammatory cytokines IL-6 and TNF-α induced by zinc deficiency [44].

### 3.9. Mechanistic Insights from Preclinical Studies

Mechanistic insights into how zinc exerts these effects were provided by Shi et al. Their research in an AR mouse model identified the p38 MAPK signaling pathway as a key target [44]. They demonstrated that zinc deficiency significantly increased the expression and activation of the p38 MAPK protein and that zinc supplementation reversed this effect. The crucial role of this pathway was confirmed experimentally; when a specific p38 inhibitor (SB203580) was administered to zinc-deficient mice, it significantly reversed the elevated levels of IgE and inflammatory cytokines (IL-6, TNF-α), thereby mimicking the therapeutic effect of zinc supplementation. These findings, as proposed by the authors, suggest that zinc exerts its beneficial effects by downregulating the p38 MAPK pathway, which in turn inhibits the production of key mediators of allergic inflammation [44].

## 4. Discussion

The findings emerging from this review highlight a complex and multifaceted relationship between zinc homeostasis and allergic airway diseases, particularly pediatric asthma and rhinitis [33]. A predominant trend in the literature suggests a significant correlation between altered zinc status and the presence of these conditions. This altered zinc status appears clinically relevant, often correlating with poorer disease control, increased disease severity, and reduced lung function [35]. The biological basis for these observations is plausible, as zinc is a crucial element for modulating immune responses, maintaining epithelial barrier integrity, and regulating oxidative stress, all of which are core components in the pathophysiology of allergic diseases [10].

### 4.1. Zinc in Asthma

A substantial body of evidence indicates that children with asthma frequently exhibit altered zinc homeostasis. Multiple systematic reviews and meta-analyses have consistently found significantly lower circulating zinc levels in asthmatic children compared to healthy controls. These findings are supported by numerous cross-sectional studies in diverse populations [34,35]. However, this evidence is not entirely uniform, as some studies have reported no significant difference in serum zinc concentrations, highlighting the high heterogeneity across research [33]. This variability is likely due to differences in study design, population characteristics, and methodologies for zinc assessment. Notably, hair zinc levels, a marker of long-term status, were consistently found to be lower in asthmatic children, suggesting that this may be a more stable biomarker than serum zinc, which can be influenced by acute inflammation [33,48]. Functionally, zinc status appears linked to clinical outcomes. Several studies have documented a positive correlation between higher serum zinc levels and better asthma control, as well as improved pulmonary function, including FEV_1_ and FEV_1_/FVC ratios [40]. Interventional studies further bolster zinc’s therapeutic potential. A randomized controlled trial demonstrated that zinc supplementation for children hospitalized with an acute asthma exacerbation significantly accelerated clinical improvement compared to a placebo [41]. Furthermore, epidemiological data from the NHANES cohort revealed an inverse association between higher dietary zinc intake and asthma prevalence among overweight or obese youths. Mechanistically, zinc deficiency is known to promote a Th2-dominant immune response characterized by elevated IgE and eosinophilic inflammation, which are hallmarks of allergic asthma [42].

### 4.2. Zinc in Rhinitis

The role of zinc in rhinitis is characterized by a unique paradox in its local distribution [43]. While studies have documented lower systemic zinc levels in patients with seasonal allergic rhinitis during the pollen season, a fascinating counter phenomenon occurs at the site of inflammation [43]. Both human and animal studies show that zinc concentrations are paradoxically increased in nasal secretions and epithelial lining fluid during active allergic reactions. This fact suggests a dynamic local shift of zinc from the systemic circulation and mucosal tissue into the airway lumen, possibly as part of a dysregulated host defense mechanism. In CRSwNP, a condition also driven by type 2 inflammation, studies have found significantly depleted zinc levels within the nasal mucosa itself [47]. This mucosal zinc depletion is clinically significant, as it correlates with key pathophysiological features of the disease, including a higher number of infiltrating eosinophils and reduced collagen content, suggesting a role in adverse tissue remodeling. Mechanistically, it has been proposed that a “vicious cycle” may occur in CRSwNP, where inflammation drives mucus hypersecretion, and zinc-binding mucins in the mucus then sequester zinc, leading to its depletion from the tissue and further exacerbating local inflammation [47]. Preclinical studies also suggest that zinc’s anti-allergic effects may be mediated, in part, through the downregulation of the p38 MAPK inflammatory signaling pathway, which is critical for producing IgE and pro-inflammatory cytokines [44].

### 4.3. Clinical Implications in Asthma and Rhinitis

Given zinc’s safe profile, low cost, and multifaceted biological roles, integrating zinc assessment and repletion strategies into clinical practice merits consideration [3]. Firstly, zinc status could serve as a potential biomarker for disease severity or control [40]. However, its application is complex [39]. The unreliability of serum zinc as a standalone marker due to its redistribution during inflammation limits its diagnostic utility. As noted, markers of long-term status, such as hair zinc, may offer a more stable alternative [3,33]. Secondly, zinc occupies a central nexus between nutrition, oxidative balance, immunoregulation, and epithelial integrity in pediatric respiratory and allergic diseases [17]. As the global burden of asthma and atopy continues to escalate, harnessing micronutrient interventions, such as zinc supplementation, may offer a cost-effective and low-risk means to enhance disease control, reduce exacerbations, and improve the quality of life for affected children. For rhinitis, preclinical data are compelling, showing that both oral and direct intranasal zinc application can effectively alleviate allergic symptoms and reduce inflammatory markers in animal models [43]. The potential for developing a topical, intranasal zinc therapy that acts directly at the site of inflammation is particularly intriguing. However, a critical step is to identify the patient subgroups most likely to benefit, such as those with confirmed zinc deficiency or specific disease phenotypes, like severe eosinophilic asthma or CRSwNP [47]. In conclusion, targeted evaluation of zinc status in atopic children may offer clinical utility. In high-risk groups, identifying zinc deficiency could reveal a modifiable factor contributing to airway inflammation and disease severity, potentially supporting the use of standardized supplementation strategies as part of a more personalized treatment approach.

### 4.4. Limitations of Current Studies

Before these clinical implications can be fully realized, it is essential to acknowledge the limitations of the current body of evidence. A major issue across the literature is the significant heterogeneity in study results, particularly in meta-analyses of serum zinc levels. This fact is likely due to a lack of standardization in study design, population characteristics, zinc assessment methodologies, and inadequate control for confounding factors such as dietary patterns and corticosteroid use. In interventional trials, additional methodological weaknesses further limit the strength of the conclusions. Sample sizes are frequently small, reducing statistical power and generalizability. Several studies lack blinding or randomization, introducing risk of bias. Zinc dosage, formulation, route of administration (oral vs. intranasal), and treatment duration vary considerably across studies. Moreover, outcome measures are inconsistent, ranging from symptom scores to diverse biochemical endpoints, with limited standardization. Control groups are often poorly described or heterogeneous. Furthermore, the predominance of observational, cross-sectional studies makes it difficult to establish causality; it remains unclear whether low zinc is a cause or a consequence of chronic inflammation. A Mendelian randomization study designed to address this issue did not find a causal link between genetically predicted serum zinc levels and the risk of allergic rhinitis, highlighting the complexity of the relationship [45]. However, the same study reported inverse associations between genetically predicted serum zinc levels and the risk of allergic asthma and atopic dermatitis, suggesting that zinc’s immunomodulatory effects may vary across different allergic disease phenotypes. These findings reinforce the idea that zinc may play a disease-specific role in immune regulation and that the absence of causality in allergic rhinitis does not preclude potential benefits in other allergic conditions. Furthermore, many studies do not adequately control for potential confounding factors, such as dietary patterns, socioeconomic status, and the use of medications like corticosteroids, which could independently influence both zinc levels and disease outcomes.

### 4.5. Future Perspectives and Research Directions

To move the field forward, a multi-pronged research approach is necessary. There is a clear need for large-scale, long-term, randomized controlled trials in human subjects to definitively establish the efficacy and safety of zinc supplementation for both asthma and rhinitis. Such trials should incorporate standardized protocols for assessing zinc status (including markers beyond serum zinc), dosage, and formulation. They should evaluate clinically meaningful long-term outcomes, such as exacerbation frequency, decline in lung function, and quality of life. Future research should also embrace a “personalized medicine” approach, aiming to identify which patients benefit most. This fact could involve stratifying participants by baseline zinc status, genetic polymorphisms in zinc transporters, or disease endotype (e.g., Th2-high vs. non-Th2). The promising preclinical results for intranasal zinc delivery warrant dedicated clinical trials to assess its efficacy and safety in humans as a novel topical therapy for AR. Furthermore, given the frequent co-occurrence of deficiencies in other immunomodulatory micronutrients, such as selenium and vitamin D, future interventional studies should consider exploring synergistic, multi-nutrient formulas rather than focusing solely on zinc. While this review deliberately focused on zinc due to its well-established role in immune modulation, antioxidant defense, and epithelial integrity, emerging evidence suggests that its biological effects may be potentiated when combined with other trace elements. Selenium, for example, acts in tandem with zinc to regulate oxidative stress and inflammatory responses through the activity of selenoproteins and zinc-dependent enzymes. Vitamin D, on the other hand, plays a complementary role in maintaining mucosal barrier integrity and modulating innate and adaptive immunity, with possible additive or synergistic effects on T-cell polarization and cytokine regulation. These combined actions may enhance the efficacy of supplementation strategies, particularly in children with complex micronutrient imbalances or chronic allergic inflammation. Finally, investigating the intricate relationship between zinc, the gut and airway microbiome, and epithelial barrier function could uncover novel mechanisms and provide a more holistic understanding of how nutrition modulates allergic airway disease.

## 5. Conclusions

Zinc emerges as a pivotal micronutrient in the pathophysiology of pediatric allergic airway diseases, particularly asthma and allergic rhinitis. Current evidence indicates that altered zinc homeostasis, most often manifesting as a deficiency, is associated with poorer disease control, increased symptom severity, and impaired lung function in asthmatic children. Similarly, in allergic rhinitis, zinc depletion at the tissue level correlates with key inflammatory features, including eosinophilic infiltration and dysfunction of the epithelial barrier. Mechanistically, zinc plays a multifaceted role by modulating oxidative stress, preserving epithelial integrity, and regulating both innate and adaptive immune responses. These biological effects underpin the therapeutic potential of zinc supplementation, which has demonstrated improvements in clinical symptoms, inflammatory markers, and pulmonary parameters in several pediatric studies, particularly among those with documented deficiency. Despite these promising findings, substantial heterogeneity in study design, assessment methods, and outcome measures limit the strength of current recommendations. Moreover, the predominantly observational nature of the evidence and the lack of standardized supplementation protocols preclude definitive conclusions about causality and clinical efficacy. Nevertheless, given its safety profile, low cost, and biological plausibility, zinc represents a compelling adjunctive strategy in the management of pediatric asthma and allergic rhinitis. Future research should prioritize large-scale, randomized controlled trials with standardized methodologies to determine optimal dosing, identify responder subgroups, and evaluate long-term outcomes. Integrating zinc status assessment into routine clinical evaluation, especially in children with severe or poorly controlled allergic disease, may offer a pathway toward more personalized and effective treatment strategies.

## Figures and Tables

**Table 1:** Comprehensive summary of key studies on zinc in asthma and rhinitis.

First Author (Year)	Study Type	Population/Model	Zinc Assessment Method	Zinc Levels (Patient Group vs. Control Group)	Key Findings & Conclusion
Asthma Studies					
Maywald, M. (2024) [3]	Review	Allergic diseases	N/A	N/A	Zinc deficiency promotes a Th2-dominant immune status and impairs epithelial barriers, contributing to allergy development.
Peroni, D.G. (2023) [10]	Review	Atopic diseases	N/A	N/A	Posits that deficiencies of zinc, iron, and vitamins are key contributors to the etiology of atopic diseases by skewing immune responses.
Rajkumar, S. (2023) [27]	Cross-sectional	67 children with asthma (6–18 years)	Serum; Photometry	Controlled: 158.06 µg/dL; Uncontrolled: 129.23 µg/dL (*p* = 0.006)	Serum zinc is significantly higher in controlled asthma. A weak positive correlation exists between zinc levels and asthma control scores.
Ghaffari, J. (2021) [33]	Systematic Review and Meta-analysis	21 articles (pediatric asthma)	Serum, Hair, Nail, Erythrocyte	Serum: No significant difference (pooled data); Hair: Consistently lower in asthmatics	While serum zinc levels are inconsistent, hair zinc may be a better marker. Zinc supplementation appears to improve clinical symptoms.
Chen, M. (2020) [34]	Meta-analysis	26 studies	Circulating (Serum/Plasma)	Effect Size: SMD = −0.40 (95% CI: −0.77 to −0.03)	Asthma patients have significantly lower circulating zinc levels compared to healthy controls.
Xue, M. (2024) [35]	Meta-analysis	21 articles (2205 children)	Circulating (Serum/Plasma)	Effect Size (Asthma): SMD = −0.41 (95% CI: −0.65 to −0.16)	Lower circulating zinc is significantly associated with an increased risk of childhood asthma.
Kuti, B.P. (2020) [36]	Cross-sectional	80 asthmatic vs. 80 control children (Nigeria)	Serum	Asthmatics: 71.0 ± 30.3 µg/dL; Controls: 84.2 ± 31.7 µg/dL (*p* = 0.008)	Asthmatic children have significantly lower serum zinc levels, but no association was found between this and disease severity or symptom control.
Srivastava, S. (2023) [37]	Cross-sectional	100 asthmatic children (avg. 8.7 yrs) vs. 75 controls	Serum	Asthmatics: 51 ± 12.8 µg/dL; Controls: 60 ± 18.2 µg/dL (*p* = 0.0002)	Asthmatic children have significantly lower serum levels of zinc, selenium, and vitamin D3. Low zinc is associated with poorer asthma control.
Andino, D. (2019) [38]	Case–control	12 children with moderate–severe persistent asthma vs. 12 controls	Serum	Asthmatics: 759 µg/L (median); Controls: 910 µg/L (median) (*p* = 0.011)	Asthmatics have statistically lower serum zinc levels, though not meeting the threshold for clinical deficiency.
AbdulWahab, A. (2018) [39]	Cross-sectional	40 asthmatic vs. 40 control children (Qatar)	Serum	Asthmatics: 12.78 ± 1.8 µmol/L; Controls: 13.0 ± 1.52 µmol/L (*p* > 0.05)	No significant difference in serum zinc levels between groups. No association was found between zinc and asthma control.
Siripornpanich, S. (2021) [40]	Cross-sectional	76 children with persistent asthma (Thailand)	Plasma; Atomic Absorption Spectrophotometry	Mean: 54.1 µg/dL (all participants below normal range)	All participants were zinc deficient. Plasma zinc positively correlated with lung function (FEV1 and FEV1/FVC ratio).
Rerksuppaphol, S. (2016) [41]	RCT	42 children with acute asthma exacerbation	Serum	Baseline (avg.): 63.8 µg/dL in both groups	Zinc supplementation (30 mg/day) significantly accelerated clinical improvement (PRAM score) at 24 and 48 h.
Cheng, C. (2024) [42]	Cross-sectional (NHANES)	4597 overweight/obese children and adolescents	Dietary Recall (24 h)	Intake assessed by quartiles (mg/day)	Higher dietary zinc intake is inversely associated with asthma prevalence in a dose-response manner (OR = 0.71 for Q4 vs. Q1).
Rhinitis Studies					
Suzuki, M. (2020) [28]	Human Study	CRS patients vs. controls	Serum, Mucus, Mucosal Tissue	Serum: No difference; Mucus: Increased in inflamed sites; Tissue: Decreased in CRSwNP	Mucosal tissue zinc depletion in CRSwNP correlates with eosinophilia and collagen depletion, suggesting a role in pathophysiology.
Xu, H. (2025) [43]	Human Observational and Animal Study	44 AR patients vs. 57 controls and mouse model	Serum, Nasal Epithelial Lining Fluid (ELF)	Serum: Decreased in patients; Nasal ELF: Increased in patients	Paradoxical zinc distribution found during allergic inflammation. Intranasal zinc application alleviates allergic symptoms in mice.
Changhai, L. (2025) [45]	Mendelian Randomization	General population (European ancestry)	Genetic Proxies (from GWAS)	N/A	No evidence was found for a causal association between genetically predicted serum zinc levels and the risk of allergic rhinitis.
Shi, Q. (2023) [44]	Animal Study	OVA-induced allergic rhinitis mouse model	N/A	N/A	Zinc supplementation reverses high IgE and inflammatory cytokines by downregulating the p38 MAPK pathway.
